# Crystallographic Structure of Human Dihydroorotate Dehydrogenase in Complex with the Natural Product Inhibitor Lapachol

**DOI:** 10.1021/acsomega.5c01536

**Published:** 2025-07-03

**Authors:** Aline D. Purificação, Laila S. Benz, Wemenes J. Lima Silva, Flavio S. Emery, Carolina Horta Andrade, Manfred S. Weiss, Maria Cristina Nonato

**Affiliations:** † Center for the Research and Advancement in Fragments and Molecular Targets (CRAFT), School of Pharmaceutical Sciences at Ribeirao Preto, 67782 University of São Paulo, Ribeirão Preto 14040-903, São Paulo, Brazil; ‡ Protein Crystallography Laboratory, Department of Biomolecular Sciences, School of Pharmaceutical Sciences at Ribeirao Preto, University of São Paulo, Ribeirão Preto 14040-903, São Paulo, Brazil; § Institut für Chemie und Biochemie, Freie Universität Berlin, Thielallee 63, 14195 Berlin, Germany; ∥ Macromolecular Crystallography, 28340 Helmholtz-Zentrum Berlin, Albert-Einstein-Straße 15, 12489 Berlin, Germany; ⊥ Laboratory for Molecular Modeling and Drug Design (LabMol), Faculty of Pharmacy, 67824 Universidade Federal de Goiás, Goiânia 74605-170, Goiás, Brazil; # Center for Excellence in Artificial Intelligence (CEIA), Institute of Informatics, Universidade Federal de Goiás, Goiânia 74605-170, Goiás, Brazil

## Abstract

Dihydroorotate dehydrogenase (DHODH) is a key enzyme in the pyrimidine biosynthesis pathway, playing a critical role in cellular processes and offering therapeutic potential for antiviral, antineoplastic, and autoimmune treatments. Human DHODH (*Hs*DHODH) utilizes ubiquinone as a second substrate, positioning its quinone-binding site as a promising target for inhibitor development. Lapachol, a natural naphthoquinone, has gained prominence as a valuable natural product for the discovery of novel therapeutic agents, thanks to its wide range of biological activities. In this study, we present the first crystal structure of *Hs*DHODH in complex with lapachol, providing valuable insights into the interactions between this natural product and the enzyme. The structure reveals key binding interactions that mediate lapachol’s affinity for *Hs*DHODH and validates previously proposed computational models. Complementary molecular dynamics simulations further highlight the stability of the complex and the importance of water-mediated interactions in ligand binding. These findings enhance our understanding of how naphthoquinone derivatives, such as lapachol, interact with class 2 DHODHs, offering a foundation for the design of optimized inhibitors for therapeutic applications. By integration of structural and computational data, this study contributes to the rational design of novel *Hs*DHODH inhibitors, paving the way for future exploration of lapachol and its derivatives in drug discovery.

## Graphical abstract

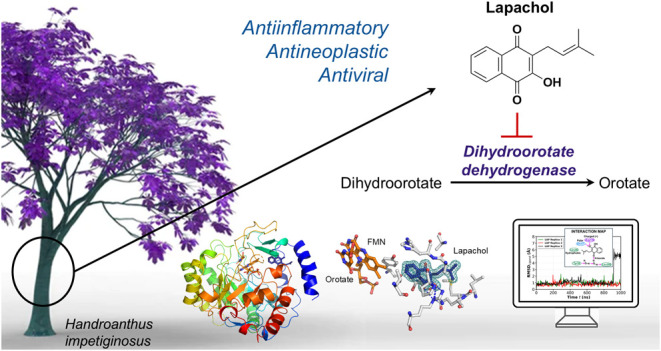

## 1 Introduction

Dihydroorotate dehydrogenase (DHODH) is a key enzyme in the pyrimidine biosynthesis pathway, essential for DNA and RNA syntheses in all organisms.^1^ Human DHODH (*Hs*DHODH) belongs to class 2 DHODHs, which occur in the mitochondrial membrane and operate through a ping-pong reaction mechanism involving two distinct steps. In the first step, dihydroorotate (DHO) is oxidized to orotate (ORO) while flavin mononucleotide (FMN) is reduced. In the second step, the reduced FMN is reoxidized by a second substrate, varying by organism, ensuring the continuous catalytic cycle of the enzyme.^2^ In *Hs*DHODH, the second substrate is ubiquinone, which is reduced to ubiquinol.

The inhibition of DHODH in various organisms is linked to antiparasitic, antineoplastic, and antiviral activities. *Hs*DHODH is a significant therapeutic target for diseases such as cancer,^3^ autoimmune disorders,^4^ and viral infections.^5^ Host-targeted antiviral therapy, a modern approach, utilizes *Hs*DHODH inhibition to reduce the intracellular nucleotide pool, impairing viral replication, as viruses rely on host nucleotides for rapid reproduction.

Teriflunomide, the active metabolite of leflunomide, is a clinically approved *Hs*DHODH inhibitor used to treat rheumatoid arthritis.^6^ Several other inhibitors have been described, some of which are undergoing clinical trials for cancer and viral infections (NCT04997993,^7^ NCT04575038 ^8^).

Among natural inhibitors of *Hs*DHODH, lapachol, a 1,4-naphthoquinone derivative extracted from *Tabebuia impetiginosa*, has been studied since the 19th century for its antineoplastic, antibiotic, and antimalarial properties.^9,10^ Naphthoquinone derivatives, such as lapachol, demonstrate anticancer activities through various mechanisms, including topoisomerase inhibition, modulation of the tumor suppressor p53, and inhibition of MALT.^11^ Recently, we have identified lapachol as a potent inhibitor of both *Hs*DHODH and DHODH of *Schistosoma mansoni* (*Sm*DHODH).^12^ These class 2 DHODHs are monomeric proteins anchored to cellular membranes and require quinones as their physiological oxidizing agent. Given the biological role of quinones in the activity of *Hs*DHODH and *Sm*DHODH, it is anticipated that quinone derivatives like lapachol will interact effectively with these enzymes.

Moreover, previous docking studies suggested the interactions between lapachol and *Hs*DHODH,^5^ as well as between lapachol and *Sm*DHODH^13^ on the same binding site, that is partially conserved between class 2 DHODHs. However, these studies were limited by the absence of experimental structures of lapachol in complex with class 2 DHODHs, relying solely on computational models.^5,13^

To date, no crystallographic data has been available for lapachol in complex with any class 2 DHODH, despite extensive interest in this compound’s biological activity. The lack of a crystal structure in complex with lapachol, potentially linked to the low solubility of this compound (log *P* = 2.8),^14^ has limited the validation of these computational models and the detailed understanding of the key interactions that underlie the potency and selectivity of lapachol and its derivatives. Our structure fills this gap by providing, for the first time, experimental confirmation of Lapachol’s binding mode on *Hs*DHODH, validating previous docking studies, together with dynamic simulations that highlight the stability of the ligand at the binding site and at the same time expose the stability of a couple of waters at the binding site that may impact ligand affinity.

## 2 Results and Discussion

The *Hs*DHODH–lapachol complex was solved at 1.31 Å, comprising 365 residues (Thr^32^ to Arg^396^), 1 FMN molecule, 1 ORO molecule, 1 acetic acid molecule, 3 glycerol molecules, 3 sulfate ions, 1 lapachol molecule, and 245 water molecules treated as oxygens. The coordinates for this structure have been deposited in the PDB under accession code 9EG9. Data collection and structure refinement statistics are summarized in Tables 2 and 3. The overall structure reveals the characteristic α-β barrel fold typical of *Hs*DHODH structures (Figure 1). The high-resolution structure exhibits excellent geometric quality and a strong fit to the electron density (Figure S2), with the main challenges observed in the N-terminal region, consistent with findings in other *Hs*DHODH structures.^15,16^

**Figure 1:**
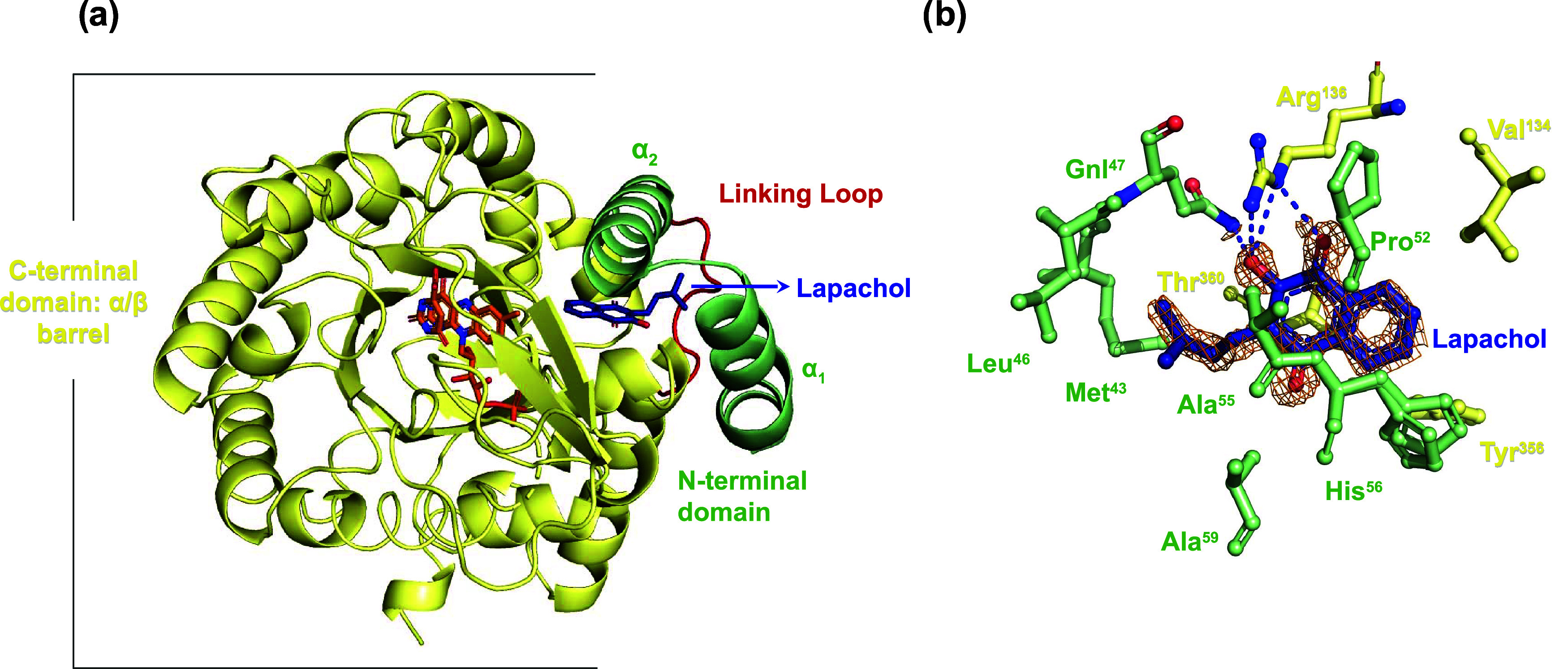
Structural overview of *Hs*DHODH–lapachol and the binding site. (a) Cartoon representation of the overall fold, showing the C-terminal domain (Met^78^-Arg^396^), the linking loop, and the N-terminal domain α_1_ and α_2_ helices (Met^30^-Leu^68^). Orotate and FMN are orange, and lapachol is blue. The C-terminal domain is colored yellow, the N-terminal domain is colored green, and the linking loop is colored red. (b) Close-up view of the lapachol binding site in the crystal structure with hydrogen bonds depicted as blue dotted lines. The inhibitor and residues involved in hydrogen bonding are colored by atoms: oxygen (red), nitrogen (blue), and carbon (pale green for N-terminal residues, yellow for C-terminal residues, and blue for lapachol). The omit map (contoured at 3 RMSD) is shown around the ligand.

A region of particular interest is the loop comprising residues Asn^212^ to Leu^224^, which has been implicated in mediating the orotate release mechanism.^15^ While this loop is often disordered in *Hs*DHODH structures complexed with inhibitors, in the *Hs*DHODH–lapachol complex, it is well-defined and can be reliably modeled.

Lapachol binds to the N-terminal region of the enzyme, occupying the proposed ubiquinone-binding site where all currently described *Hs*DHODH inhibitors are bound.^2^ This study discloses the first crystal structure of *Hs*DHODH in complex with a quinone, providing strong evidence that this site is indeed the binding region for the second substrate, ubiquinone. The benzoquinone ring of ubiquinone contains redox-active sites, whereas the polyisoprenoid chain is responsible for positioning the molecule within the midplane of the lipid bilayer of various cell membranes.^17^ In the case of *Hs*DHODH, the long polyisoprenoid chain of ubiquinol likely interacts with the enzyme’s transmembrane domain, which anchors it to the mitochondrial membrane. Notably, the quinoidal core of ubiquinone closely resembles that of lapachol, while the branched tail of lapachol mirrors the polyisoprene chain of ubiquinone, albeit with a shorter length (10 units in ubiquinone). These structural similarities lend support to a plausible mechanism of the ubiquinone interaction in this binding site.

This pocket is also the binding site for brequinar, one of the most well-characterized *Hs*DHODH inhibitors, extensively studied for potential applications in cancer,^3,16^ infectious diseases,^5,18^ and inflammatory disorders.^19^ In the crystallographic structure reported here, the hydroxyl group attached to carbon 2 and the carbonyl group attached to carbon 1 of the naphthoquinone moiety of lapachol form interactions with the side chain of Arg^136^, resembling those mediated by the carboxy group of the five-membered ring of brequinar analogue (PDB ID: 1D3G) (Figure 2). Additionally, the hydroxyl group on carbon 2 of lapachol’s naphthoquinone also interacts with the side chain of Gln^47^, an interaction similar to that of brequinar’s carboxyl group in the same region. This interaction pattern has been classified by Baumgartner et al. as brequinar-like.^20^

**Figure 2:**
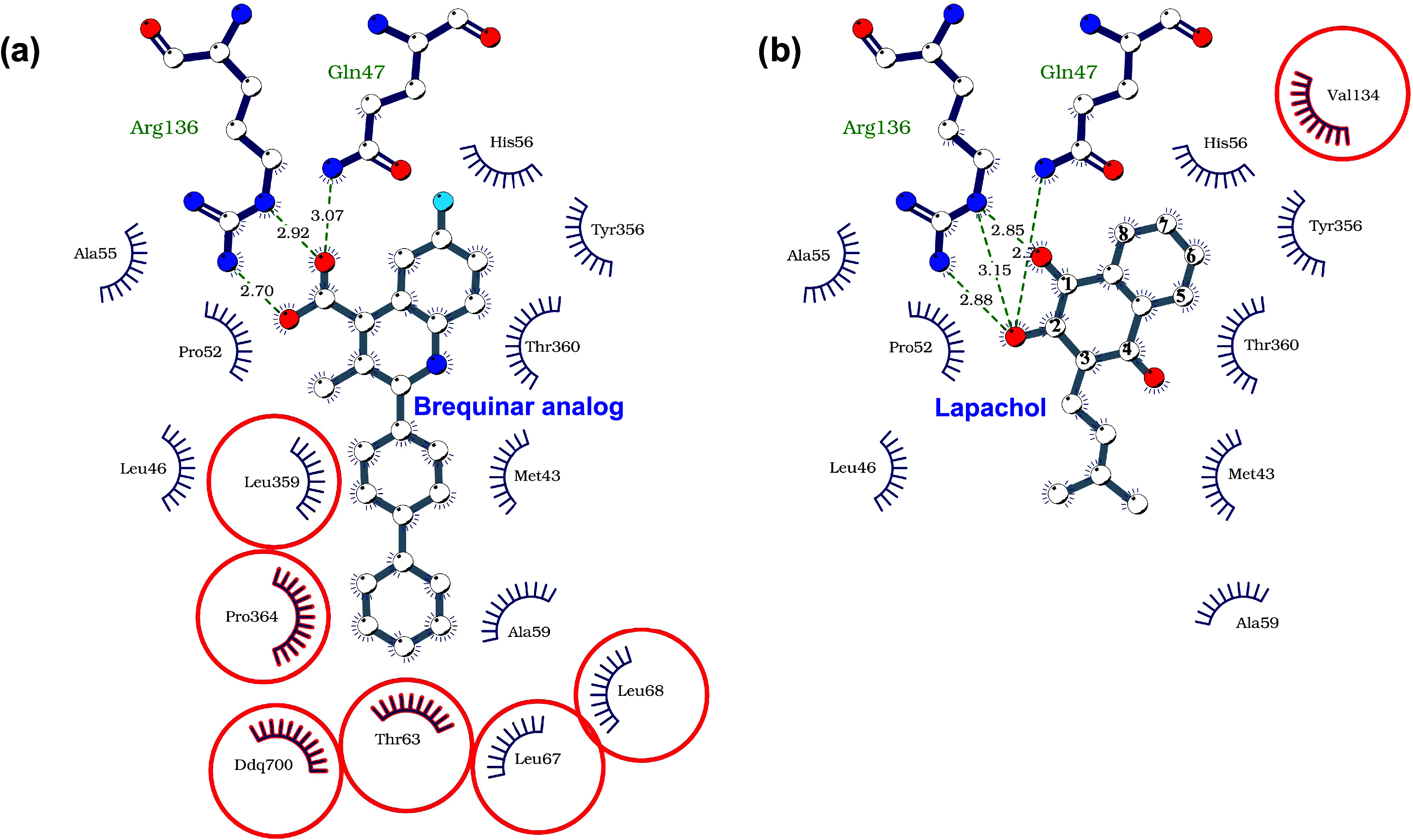
Brequinar-like interaction pattern of lapachol is confirmed by the crystal structure. Representation of the binding site that accommodates a brequinar analogue (a) and the lapachol (b), showing the hydrogen bonds as dotted green lines and the residues responsible for hydrophobic interactions. The plot was generated by LigPlus using the crystallographic structures of *Hs*DHODH in complex with a brequinar analogue (PDBID 1D3G) and lapachol. The residues in red highlight the differences in the interaction patterns, as they are residues that interact solely with the brequinar analogue (a) or lapachol (b).

According to Baumgartner’s classification, lapachol interacts with subsites 1 (Met^43^, Leu^46^, Ala^55^), 2 (Arg^136^, Gln^47^), 3 (Tyr^356^), and 4 (Val^134^). Additionally, lapachol interacts with Pro^52^, His^56^, Thr^360^, and Ala^59^—residues that were described to interact with brequinar but are not included in Baumgartner’s subsite classification. These findings support a “brequinar-like” binding mode for lapachol, as predicted by prior docking studies from our group^5^ (Figure 2). Notably, the interaction with Val^134^ is unique to lapachol. Brequinar interacts with residues Pro^364^, Thr^63^, Leu^67^, and Leu^68^, which may contribute to its higher potency compared to lapachol.^5^

Comparing the crystal structure of *Hs*DHODH–lapachol here presented with the docking model previously obtained by our group,^5^ we can see that the docking calculations accurately predicted the lapachol pose and the residues involved in its interactions (Figure 3). The primary difference between the predicted pose and the crystallographic structure is observed in the isoprenyl group of lapachol, which likely reflects the conformational flexibility of the isoprenyl chain and does not involve significant rearrangements of the ligand or protein side chains. In the crystal structure, the isoprenyl group interacts with Leu^46^, whereas in the predicted pose, it interacts with Leu^359^. Notably, a slight conformational change in the side chain of Arg^136^ observed in the crystal structure, with a root-mean-square deviation for all atoms (RMSD_all_) of 1.26 Å, enabled interactions with both the carbonyl group at carbon 1 and the hydroxyl group at carbon 2 of the naphthoquinone core. In contrast, the docking model predicted only the interaction with the hydroxyl group. A minor variation in the side chain of Gln^47^ (RMSD_all_ = 0.78 Å) also facilitated a hydrogen bond with the quinonoid core of lapachol in the crystal structure, a feature not predicted in the docking model.

**Figure 3:**
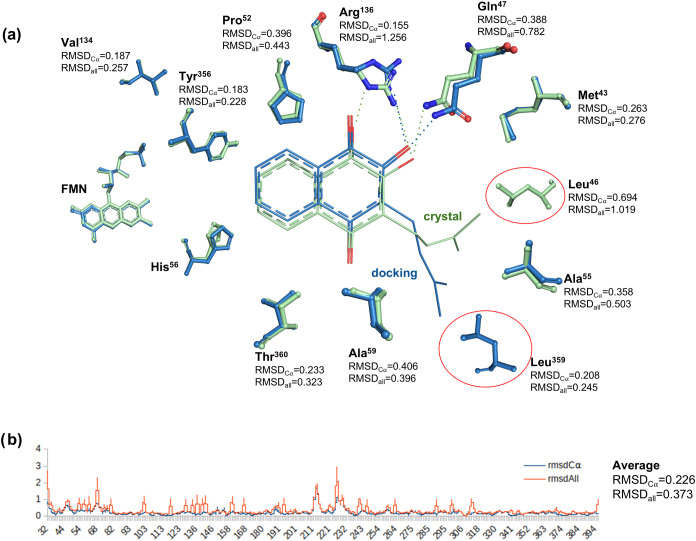
Computational prediction of the *Hs*DHODH–lapachol complex closely resembles the experimental structure. (a) Schematic representation comparing the lapachol binding site in the predicted model (blue) and the crystallographic structure (green), highlighting the ligand and the residues involved in interactions, along with their respective RMSD_Cα_ and RMSD_all_ values. The residues Leu^46^ and Leu^359^ are emphasized, as they interact with the isoprenyl portion of lapachol in different conformations observed in the docking model and the crystal structure. (b) RMSD_Cα_ and RMSD_all_ by residue plot showing RMSD_Cα_ (blue) and RMSD_all_ (orange) values on the *Y*-axis for each residue along the *X*-axis.

Overall, the predicted and experimental structures are highly similar, with the average root-mean-square deviation for Cα carbon (RMSD_Cα_) and RMSD_all_ values of 0.23 and 0.37 Å, respectively, indicating a modest deviation (Figure 3b). The maximum RMSD_Cα_ and RMSD_all_ values are also small, 1.84 and 2.95 Å, respectively, and are observed in the loop region comprising residues Asn^212^ to Leu^224^, near the reaction product, orotate. The residue interacting directly with orotate, Asn^217^, shows the lowest root-mean-square deviation values for this region (RMSD_Cα_ = 0.20 Å and RMSD_all_ = 0.22 Å), indicating high consistency between the predicted and experimental structures.

Our group previously reported that lapachol inhibits the enzyme activity of *Hs*DHODH (IC_50_ 100 ±  7 nM) and *Sm*DHODH (IC_50_ 19  ±  2 nM).^12^ To further elucidate the similarities and differences in the modes of interaction of lapachol with both enzymes, we conducted molecular docking studies. When comparing our crystallographic structure of lapachol in complex with *Hs*DHODH and our docking model of lapachol with *Sm*DHODH, it is observed that the interaction mode is similar, despite the moderate sequence identity (Figure 4). The quinoidal core of lapachol on *Sm*DHODH is flipped in relation to the position found in the crystal structure of *Hs*DHODH in complex with lapachol. This new conformation of the quinoline chain was previously described in the crystallographic structure of the *Sm*DHODH enzyme determined in complex with (2-((4-fluorophenyl)­amino)-3-hydroxynaphthalene-1,4-dione), a simplified analogue of atovaquone (PDB ID 6UY4 ^21^). The isoprenyl chain assumes a different conformation on the binding site of the homologous proteins, allowing hydrophobic interaction with Leu^46^ on the human enzyme while with Gly^351^ on the parasitic one. Some of the residues that were found to interact with lapachol in *Hs*DHODH are changed by amino acid groups of dissimilar properties in *Sm*DHODH, but with a similar interaction mode with lapachol, such as Met^43^ in *Hs*DHODH, which is replaced by Leu^36^ in *Sm*DHODH, Ala^59^ by Ser^53^, Thr^360^ by Val^358^, Val^134^ by Ile^128^, and Pro^52^ by Gly^46^. Key residues such as Arg^136^, His^56^, and Ala^55^ are conserved between the two enzymes. In addition, the interaction of lapachol with Tyr^356^ is not observed in *Sm*DHODH, although this residue is conserved between the proteins. The similarity in the interaction mode of lapachol with both enzymes makes it difficult to account for the 5-fold difference in potency for the *Sm*DHODH^12^ solely based on the structural data, highlighting the need for further studies to investigate key differences in the binding mechanism of these enzymes with lapachol and how these variations influence reaction kinetics.

**Figure 4:**
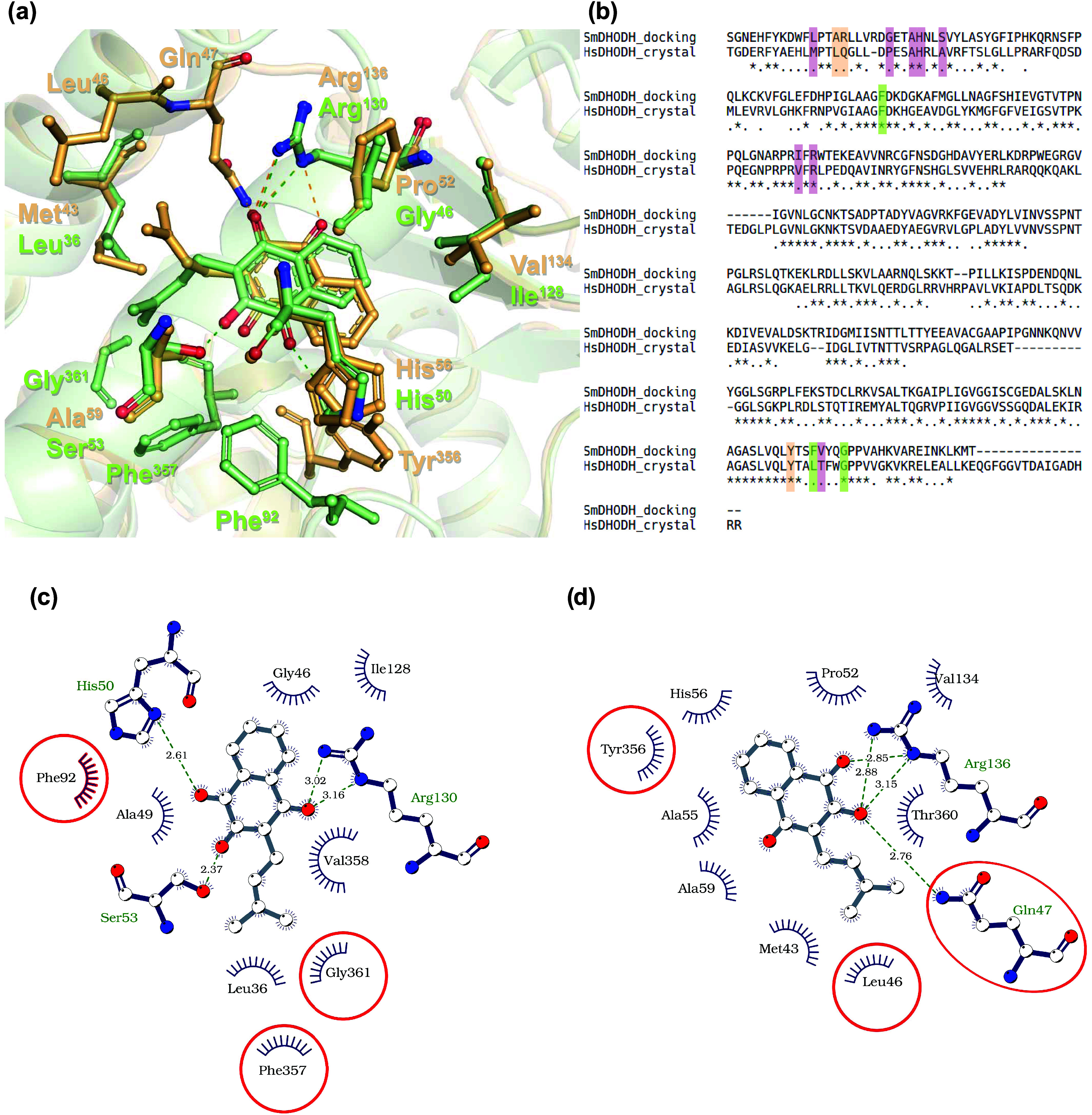
Interaction mode of lapachol with *Sm*DHODH and *Hs*DHODH. (a) A superposition of the lapachol binding site of the docking model of *Sm*DHODH (green) and the crystallographic structure of *Hs*DHODH (wheat). The protein is represented as a cartoon with transparency, while lapachol and the residues involved in the interaction are represented by sticks. (b) Structural alignment of *Hs*DHODH and *Sm*DHODH, highlighting the residues that interact only with *Sm*DHODH (green), *Hs*DHODH (wheat), or both enzymes (red). (c, d) Representation of the binding site that accommodates lapachol in *Sm*DHODH (c) and *Hs*DHODH (d), showing the hydrogen bonds as dotted green lines and the residues responsible for hydrophobic interactions. The plot was generated by LigPlus using the docking model of lapachol with *Sm*DHODH (c) and the crystallographic structure of *Hs*DHODH in complex with lapachol (d). The residues in red highlight the differences in the interaction patterns, as they are residues that interact solely with the lapachol in the *Sm*DHODH model (c) or in the *Hs*DHODH crystallographic structure (d).

Here, additionally, molecular dynamics (MD) simulations were performed to explore the binding interactions and mechanistic behavior of lapachol within the *Hs*DHODH binding site. The simulations were performed in three independent replicas, each running for 1 μs, with distinct initial geometries and velocities to ensure the variability and robustness in the results. The analyses employed root-mean-square deviation (RMSD), root-mean-square fluctuation (RMSF), and principal component analysis (PCA) to assess the results. The *Hs*DHODH backbone remained stable throughout all simulations, exhibiting RMSD values below 2 Å (Figures 5a and S3a). Lapachol maintained stable conformations with RMSD values below 2 Å in replicas 1 and 2. In replica 3, stability persisted until 900 ns, after which a conformational shift increased the RMSD to approximately 5 Å, suggesting the emergence of a potential secondary binding mode (Figures 5b and S1). To evaluate the persistence of this newly observed mode, the MD simulation for replica 3 was extended by an additional 500 ns. The secondary binding mode features an additional hydrogen bond with Thr360, distinguishing it from the usual binding mode observed in both the *Hs*DHODH–lapachol crystal structure and replicas 1 and 2 (Figures 6 and S1d). Furthermore, the conformation of this second binding mode is stable with RMSD values below 2 Å (Figure S3b). RMSF analysis showed minimal fluctuations (<2 Å) in *Hs*DHODH, except in loop regions and the N-terminal region, where variations reached up to 7 Å (Figures 5c and S3c).

**Figure 5:**
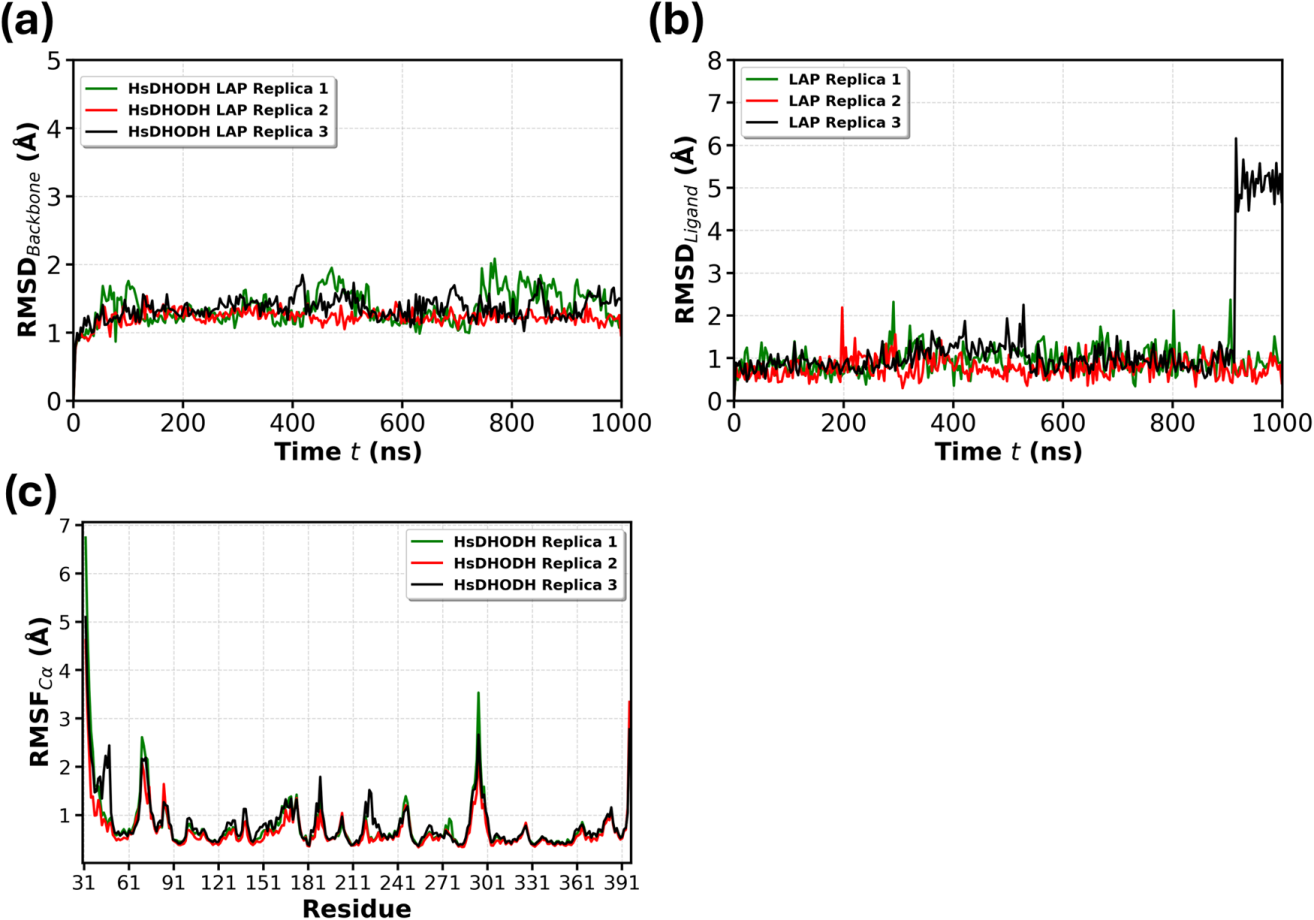
RMSD and RMSF analyses of MD simulations. (a) RMSD analysis of the *Hs*DHODH backbone protein in complex with lapachol. (b) RMSD analysis of the lapachol bound to the *Hs*DHODH binding site. (c) RMSF (Ca) analysis of the *Hs*DHODH protein in complex with lapachol.

**Figure 6:**
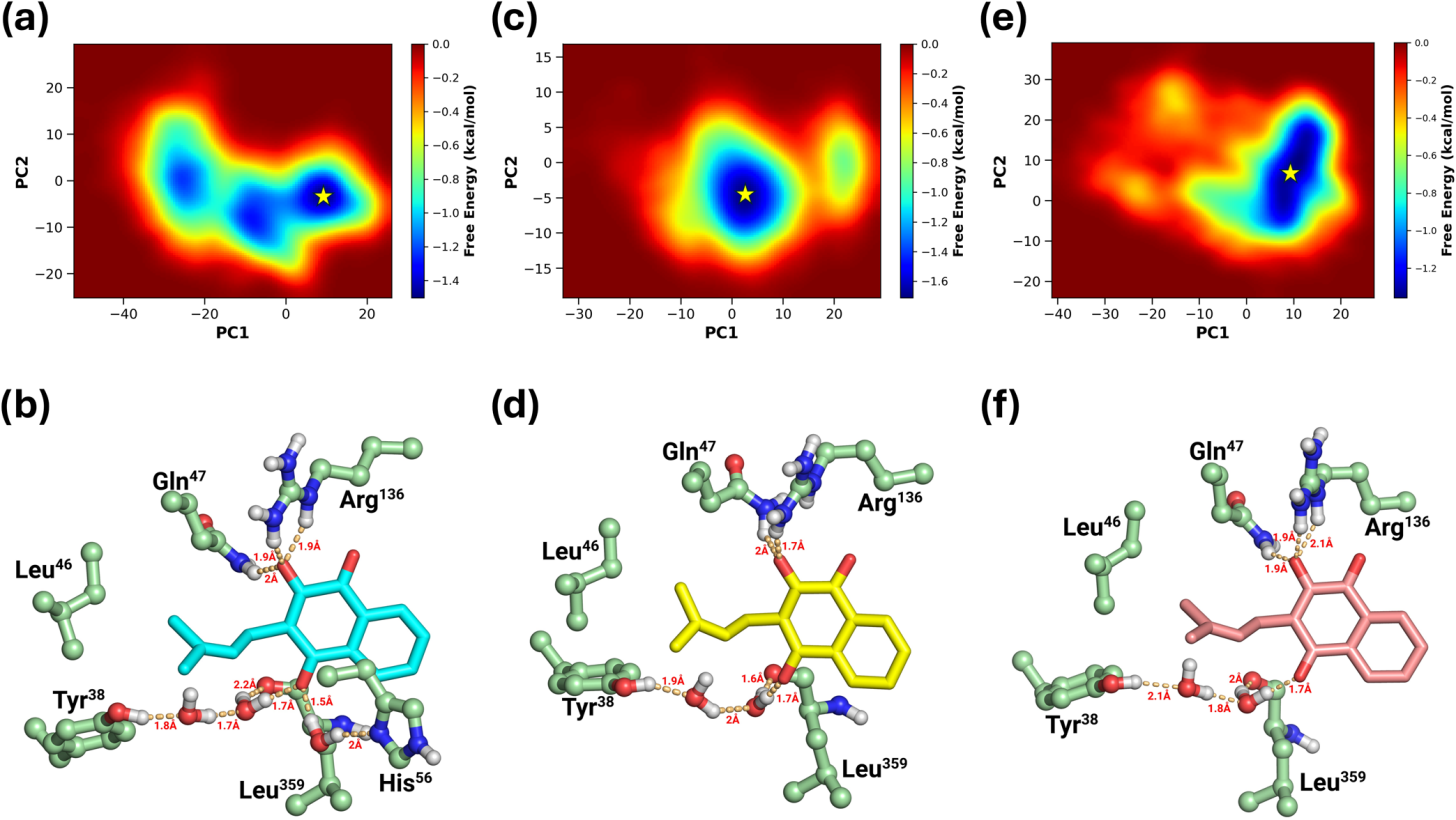
Conformational stability of the *Hs*DHODH–lapachol complex system. Free-energy landscape analysis (a, c, e) based on the first two principal components (PC1–PC2) for three replicas of the *Hs*DHODH–lapachol complex. Interaction maps illustrating the binding interactions between lapachol and the *Hs*DHODH binding site (b, d, f) are shown relative to the central frames of the free-energy basins (indicated by yellow stars). Panels (a, b) represent replica 1. Panels (c, d) represent replica 2. Panels (e, f) represent replica 3.

The free-energy landscape provides insights into the energy distribution in biomolecular systems and their conformational behavior. It describes energy barriers, stable states, and transition pathways between different molecular conformations.^22,23^ The PCA-based free-energy landscapes of the compounds provide an in-depth understanding of the conformational stability of the system (Figure 6). In replica 1, the system exhibits a free-energy basin and two regions with metastable states. In contrast, replicas 2 and 3 exhibit distinct free-energy wells (Figure 6a,c,e). Based on these free-energy landscapes, we extracted the centroid structures of the basins (Figure 6b,d,f) and used them to analyze the molecular interactions between *Hs*DHODH and lapachol.

The results demonstrated that in all three replicates lapachol forms hydrogen bonds with Arg^136^ and Gln^47^, consistent with observations in the *Hs*DHODH–lapachol crystal structure. Additionally, water-mediated hydrogen bonds were observed with residues Leu^359^, Tyr^38^, and His^56^ (Figure 6b,d,f). A secondary binding mode for lapachol, identified in replicate 3, revealed an additional hydrogen bond with Thr^360^. Furthermore, the analysis of the final frame from the extended replica 3 MD simulation revealed water-mediated hydrogen bonds among Thr^63^, Ala^55^, and Arg^136^. These findings underscore the critical role of Arg^136^ and Gln^47^ in stabilizing lapachol within the *Hs*DHODH binding site while also highlighting the importance of water-bridging interactions in ligand stabilization. Notably, although these water-mediated interactions were absent in the *Hs*DHODH–lapachol crystallographic structure, analogous interactions have been documented in other *Hs*DHODH–ligand complexes. Water-mediated hydrogen bonds with Leu^359^ and Tyr^38^ align with observations in the crystal structure of PDB ID 5ZF4.^24^ Similarly, water-mediated hydrogen bonding with His^56^ was observed in the PDB crystal ID 4OQV.^25^ As for the extended MD simulation results of replica 3, water-mediated hydrogen bonds with Ala^55^ were observed in the crystal structures PDB IDs 5HQE,^26^ 5HIN,^27^ and 5H73 ^28^ and with Arg^136^ are consistent with the crystal structures PDB IDs 6ET4,^29^ 4ZMG,^30^ 3G0X,^31^ 3FJL,^31^ 3FJ6,^31^ and 1D3H.^32^ Finally, water-mediated hydrogen bonding performed by Thr^63^ was not observed in any crystal structure. This observation suggests that solvent dynamics may play a role in modulating inhibitor stability and affinity, offering potential avenues for the rational design of derivatives that exploit these interactions.

Taking all of this structural information on lapachol into account, we propose a series of compounds that could be synthesized in further projects. As illustrated in Figure S4 (Supporting Information), polar and apolar substituents can be introduced to Ring A (electron-withdrawing or electron-donating groups) to understand the impact of stereoelectronic properties and hydrophobicity on enzyme inhibition. According to the structural analysis, it is crucial to retain the hydrogen-bonding acceptor–donor (HBA/HBD) interactions of the carbonyl-hydroxy groups in the quinoidal core (Ring B). For this purpose, we suggest either maintaining this scaffold or replacing it with heterocycles (e.g., imidazole). Additionally, we propose maintaining the hydrophobic interactions at the prenyl group position due to their importance for enzyme binding, and we also suggest substituting the carbonyl moiety adjacent to the prenyl chain with polar groups, such as oximes and oxime-ethers, or heterocycles (e.g., fused-pyrazole ring system) to mimic the water-mediated bridge of lapachol with Leu^359^ and His^56^. Future studies will be necessary to experimentally test these analogues and evaluate their potential for enzyme inhibition, antiviral activity, anti-inflammatory effects, and pharmacokinetic properties.

## 3 Conclusions

The crystallographic and dynamic structural characterization of *Hs*DHODH in complex with lapachol provides a detailed understanding of the molecular interactions underlying the inhibition of class 2 DHODHs by quinoidal compounds, such as lapachol and its derivatives. This work offers experimental validation for previously proposed computational models, enhancing our knowledge of how naphthoquinone derivatives interact with the enzyme’s binding site.

By addressing a key challenge in the study of lapachol derivatives, limited by the low solubility and the absence of structural data, this work supports ongoing efforts to develop therapeutics targeting *Hs*DHODH. Given the enzyme’s role in pyrimidine biosynthesis and its relevance to various therapeutic areas, such as antiviral and antineoplastic strategies, these findings offer practical insights for future research.

Molecular dynamics simulations provided additional insights into the stability and interaction dynamics of the *Hs*DHODH–lapachol complex. The simulations confirmed the stable binding of lapachol with interactions mediated by residues Arg^136^ and Gln^47^, consistent with the crystallographic data. The observation of water-mediated hydrogen bonds in the MD simulations, absent in the crystal structure, suggests additional avenues for exploring the influence of solvent dynamics on binding stability.

This study illustrates the importance of integrating structural and computational approaches in drug discovery. Moreover, exploring natural products, particularly lapachol, unveils the chemical and structural innovations developed by nature, providing valuable insights into modulating target protein activity for drug design. The structural data presented here enable a refined perspective on the conserved features of the binding site that facilitate quinone interactions, contributing to the rational design of improved inhibitors.

## 4 Methods

### 4.1 Protein Production and Purification

The protein was expressed and purified following an established in-house protocol^16^ using *Escherichia coli* strain BL21 Codon Plus (DE3) cells transformed with the PET28a-Sumo-*Hs*DHODH plasmid. Cells were grown in a rich medium supplemented with kanamycin and chloramphenicol, and to induce protein expression, 100 μM IPTG was added. The cultures were incubated at 18 °C for 24 h, and after expression, the cells were collected by centrifugation and disrupted by sonication.

The initial purification step involved affinity chromatography. The histidine tag was then cleaved using the ULP1 protease. For the final purification, size exclusion chromatography (SEC) was performed at pH 7.4 in a buffer containing 50 mM HEPES (pH 7.4), 400 mM NaCl, 10% (v/v) glycerol, 1 mM EDTA, and 0.05% (v/v) thesit. Fractions corresponding to the homogeneous peak obtained from SEC were analyzed using sodium dodecyl sulfate–polyacrylamide gel electrophoresis (SDS–PAGE), pooled, and concentrated to 30 mg mL^–1^. Protein concentration was determined based on a theoretical extinction coefficient (ε_280nm_) of 15.9 mM^–1^ cm^–1^ and a theoretical molecular weight of 39 887 Da.^16^

### 4.2 Protein Crystallization

Before setting up the crystallization plates, *Hs*DHODH was incubated at a final concentration of 20 mg mL^–1^ with 2 mM DHO, 20.8 mM *N*,*N*-dimethyldecylamine *N*-oxide (DDAO), and 0.75 mM lapachol for 2 h (adapted from Lewis and collaborators^16^). For crystallization, sitting drop vapor diffusion experiments were performed using 48-well MRC (SWISSCI AG) plates. Each drop, consisting of 1 μL of the protein solution mixed with 1 μL of the reservoir solution, was equilibrated against 250 μL of the reservoir solution at 20 °C. Details of the materials and methods are provided in Table 1.

**Table 1:** Details of the Crystallization Protocol

method	sitting drop vapor diffusion
plate type	MRC Maxi 48-well crystallization plate (Swissci)
temperature (K)	293.15
protein concentration	20 mg mL^–1^
buffer composition of protein solution	50 mM HEPES pH 7.4, 400 mM NaCl, 10% (v/v) glycerol, 1 mM EDTA, and 0.05% (v/v) thesit, 2 mM DHO, 20.8 mM DDAO, and 0.75 mM of lapachol
incubation time	2 h
composition of reservoir solution	0.1 M sodium acetate trihydrate pH 4.8, 1.9 M ammonium sulfate, and 30% (v/v) glycerol
volume and ratio of drop	2 μL (1:1)
volume of reservoir	250 μL

### 4.3 Data Collection and Processing

The diffraction data was collected at beamline BL14.1 (BESSY II, Berlin, Germany). The data was processed using the automatic processing pipeline XDSAPP.^33^ Data collection and processing statistics are summarized in Table 2.

**Table 2:** Details of the Data Collection and Results for Data Processing

diffraction source	beamline BL14.1 (BESSY II)
wavelength (Å)	0.9184
temperature (K)	100
detector	PILATUS3 S 6M
crystal-detector distance (mm)	151.61 mm
rotation range per image (°)	0.1°
total rotation range (°)	360°
exposure time per image (s)	0.1 s
space group	*P*3_2_21
a, b, c (Å)	90.46, 90.46, 122.66
α, β, γ (°)	90, 90, 120
resolution range (Å)	48.29–1.31 (1.39–1.31)
total no. of reflections	2780713 (448755)
no. of unique reflections	139283 (22325)
rmeas (%)	11.3 (322.5)
completeness (%)	100.0 (100.0)
redundancy	19.96 (20.1)
⟨*I*/σ(*I*)⟩	18.45 (0.98)
CC half	100.0 (41.2)

### 4.4 Structure Solution and Refinement

Initial phases were obtained by molecular replacement with PHASER^34^ using the *Hs*DHODH structure PDB code 5K9C^16^ as the search model. Model building and refinement were performed with Coot^35^ and Refmac5 ^36^ through the CCP4 suite.^37^ The quality of the final model was validated by MolProbity,^38^ and the structure was deposited in the Protein Data Bank (PDB) under accession code 9EG9. Structure refinement statistics are listed in Table 3.

**Table 3:** Results for Structure Refinement

resolution range (Å)	48.34–1.31 (1.344–1.310)
completeness (%)	99.94 (99.89)
no. of reflections, working set	132428
no. of reflections, test set	6855
final *R*_working_	0.174 (0.330)
final *R*_free_	0.180 (0.347)
no. of non-H atoms	3199
protein	2853
ligand	101
water	245
R.m.s. deviations	
bonds (Å)	0.013
angles (°)	1.65
average *B* factors (Å^2^)	
protein	21.4
ligand	28.9
water	33.9
ramachandran plot	
most favored (%)	97.8
allowed (%)	2.2

### 4.5 Molecular Docking Simulation

Molecular docking simulations were employed to analyze differences and similarities in the interaction mechanisms of the ligands 2-((4-fluorophenyl)­amino)-3-hydroxynaphthalene-1,4-dione and lapachol at the binding sites of *Sm*DHODH (PDB ID 6UY4)^21^ and *Hs*DHODH (PDB ID 9EG9), respectively. Ligands were separated from receptors, protonated using the UCSF Chimera program,^39^ and assigned AM1-BCC charges^40^ and GAFF^41^ force field parameters using the AmberTools program antechamber.^42^ The FMN cofactor was prepared following the same protocols as those applied to the ligands. Proteins were protonated, and ff14SB^43^ charges and parameters were assigned using the AmberTools program tleap. The protonation states of the ionizable residues at pH 7.4 were evaluated by means of the H++ method (http://biophysics.cs.vt.edu/H++).^44^ The assembled receptor–ligand complexes were then subjected to a short energy minimization using Amber18 in the Chimera program with strong restraints on all nonhydrogen atoms to relax the system in a controlled manner. The surface of the receptors, without ligand, was determined using DMS^45^ with a 1.4 Å probe atom radius, and the binding cavity was filled with docking beads using the DOCK6.12 tool sphgen.^46^ Finally, a docking grid was generated for each receptor with the DOCK6.12 accessory program grid^47^ within a box that surrounded all spheres with a margin of 8.0 Å in all directions and at a 0.3 Å resolution. Each grid point was assigned Lennard-Jones parameters with attractive and repulsive exponents of 6 and 9, respectively, and included a Coulombic energy term calculated using a distance-dependent dielectric constant of 4π. For redocking and cross-docking experiments, the ligands were treated as flexible based on the FLX protocol described by Mukherjee et al.^48^

### 4.6 Molecular Dynamics (MD) Simulations

MD simulations were employed to investigate the dynamic behavior of the cocrystal of *Hs*DHODH in complex with lapachol (PDB ID 9EG9). The *Hs*DHODH structure was prepared using the Protein Preparation Wizard^49^ available in the Schrödinger Suite (Schrödinger, L. Maestro Schrödinger 2021–4). We added missing atoms, adjusted side chains, and ensured accuracy of the atomic charges. Protonation and tautomeric states of amino acids were modified to match a pH of 7.4. Hydrogen bond sampling and adjustment of water molecule orientations were performed using PROPKA at pH 7.4. Structural water within 5 Å of the protein was preserved, and a minimization process with the OPLS4 force field^50^ was executed until an average root-mean-square deviation (RMSD) of 0.3 Å for the nonhydrogen atoms was achieved. In turn, the lapachol structure was assigned a protonation and ionization state at a pH of 7.4 using the LigPrep module (Schrödinger, L. Schrödinger Release 2021–4: LigPrep). Then, MD simulations were performed using the AMBER24 software package.^51^ The protein and ligand were treated with the ff14SB force field^43^ and general Amber force field version 2.2.20 (GAFF2).^52^ The complex was neutralized with a chloride ion, and then the system was solvated in TIP3P^53^ in an octahedral box 10.0 Å away from the edge. Subsequently, the system charges were neutralized with 0.15 NaCl. Then, the complex system was first minimized by the steepest descent method and the conjugate gradient method.^54^ Seven equilibration steps were applied by gradually decreasing the constraint forces. After the equilibration phase, three independent production simulations of 1 μs each were conducted for the *Hs*DHODH–lapachol complex under NPT ensemble conditions (1 atm and 310 K). To further investigate the stability of an observed conformational state, replica 3 was extended for an additional 500 ns during the production phase (totaling 1.5 μs). The Langevin thermostat^40^ was employed for temperature regulation, while the pressure was managed using the Berendsen barostat.^41^ The VMD^42^ and CPPTRAJ^43^ tools were used for trajectory analysis.

## Supplementary Material



## Data Availability

The coordinates of the model are available in the Protein Data Bank (PDB) under accession code 9EG9. The trajectories of molecular dynamics simulations are available at https://zenodo.org/records/14267492.^55^

## Notes

Lapachol secondary binding mode identified by MD showing interactions; RMSD and RMSF analyses of MD simulations for replicate 3; stereoview of the lapachol binding site in the crystal structure; and proposed modifications of the lapachol molecule that could be synthesized in further projects (PDF)
