# Supplementary material for: Crystallographic Structure of Human Dihydroorotate Dehydrogenase in Complex with the Natural Product Inhibitor Lapachol
Source: ACS Omega. 2025 Jul 3;10(27):29087–97. doi: 10.1021/acsomega.5c01536 (PMC12268739; doi:10.1021/acsomega.5c01536)
Supplement: Supplementary file 1 [file ao5c01536_si_001.pdf]

SUPPLEMENTARY MATERIAL

Crystallographic Structure of Human Dihydroorotate Dehydrogenase in Complex with the Natural Product Inhibitor Lapachol

Aline D. Purificação[1,2], Laila S. Benz[3,4], Wemenes J. Lima Silva[1,5], Flavio S. Emery[1], Carolina Horta Andrade[1,5,6], Manfred S. Weiss[4] and Maria Cristina Nonato[1,2*]

[1] Center for the Research and Advancement in Fragments and molecular Targets (CRAFT), School of Pharmaceutical Sciences at Ribeirao Preto, University of São Paulo, Ribeirão Preto 14040-903, SP, Brazil

[2] Protein Crystallography Laboratory, Department of Biomolecular Sciences, School of Pharmaceutical Sciences at Ribeirao Preto, University of São Paulo, Ribeirão Preto 14040-903, SP, Brazil

[3] Institut für Chemie und Biochemie, Freie Universität Berlin, Thielallee 63, 14195 Berlin, Germany

[4] Macromolecular Crystallography, Helmholtz-Zentrum Berlin, Albert-Einstein-Straße 15, 12489 Berlin, Germany

[5] Laboratory for Molecular Modeling and Drug Design (LabMol), Faculty of Pharmacy, Universidade Federal de Goiás, Goiânia 74605-170, GO, Brazil

[6] Center for Excellence in Artificial Intelligence (CEIA), Institute of Informatics, Universidade Federal de Goiás, Goiânia, Goiás 74605-170, Brazil.

*Correspondence email: cristy@fcfrp.usp.br

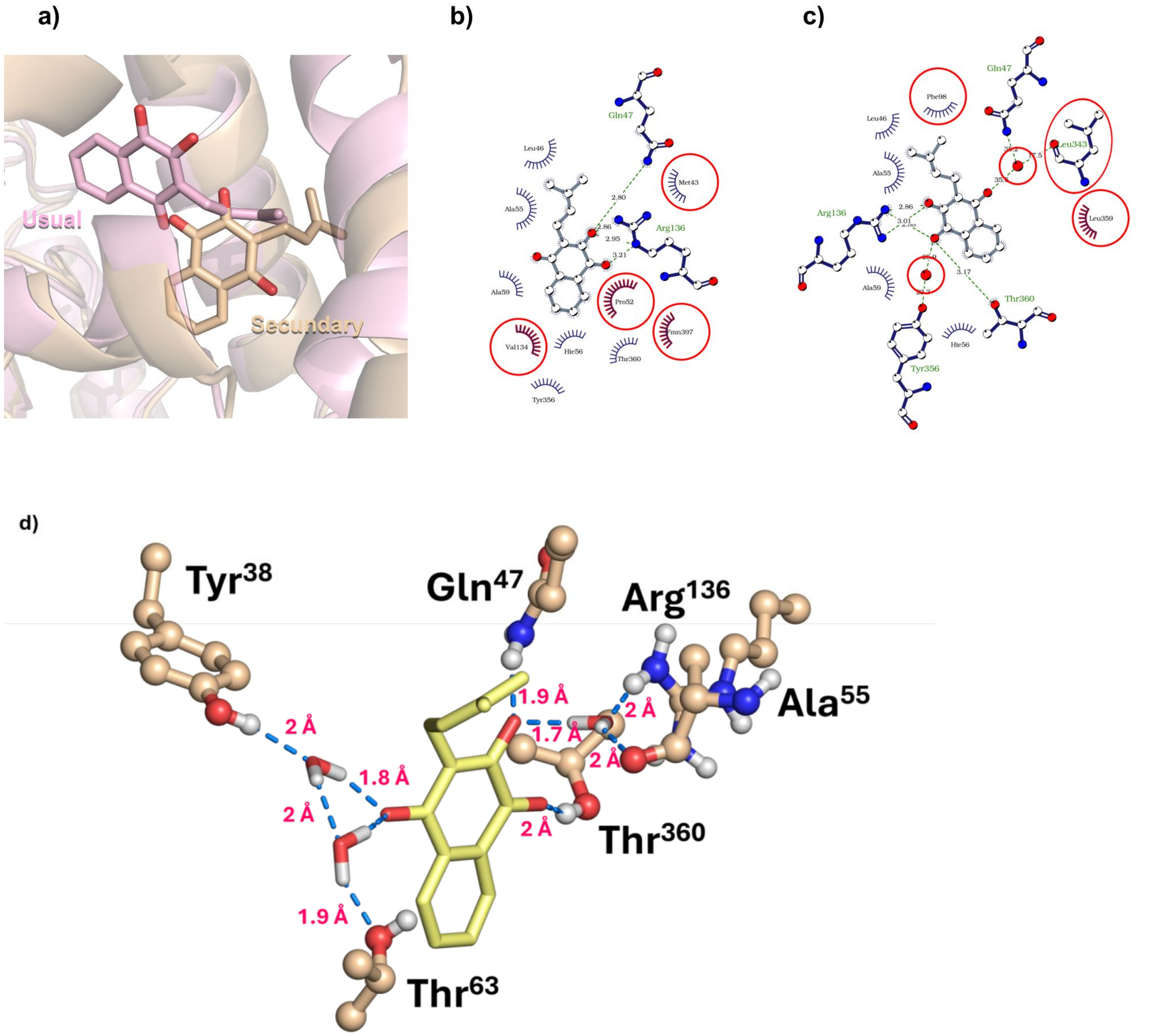

Supplementary Figure S1: Lapachol secondary binding mode. a) superposition of the representative *Hs*DHODH-complex stable during all MD simulations (pink) and secondary binding mode encountered in the final frames of replica 3 MD. b-c) Representation of the binding site that accommodates the representative *Hs*DHODH-complex stable during all MD simulations (b) and secondary binding mode encountered in the final frames of replica 3 MD (c), showing the hydrogen bonds as dotted green lines and the residues responsible for hydrophobic interactions. The plot was generated by LigPlus using the central frames of the free energy basins (b) and the final frame of replica 3 (c). The residues in red highlight the differences in the interaction patterns, as they are residues that interact solely with the lapachol in one of the conformations. (d) Interaction map of the last frame of the molecular dynamics simulation of replicate 3 after extended MD illustrating the interactions between lapachol and the amino acid residues of the HsDHODH binding sites.

Hydrogen bonds and water-mediated hydrogen bonds are illustrated in dotted blue lines and distances in red and measured in Angstroms (Å).

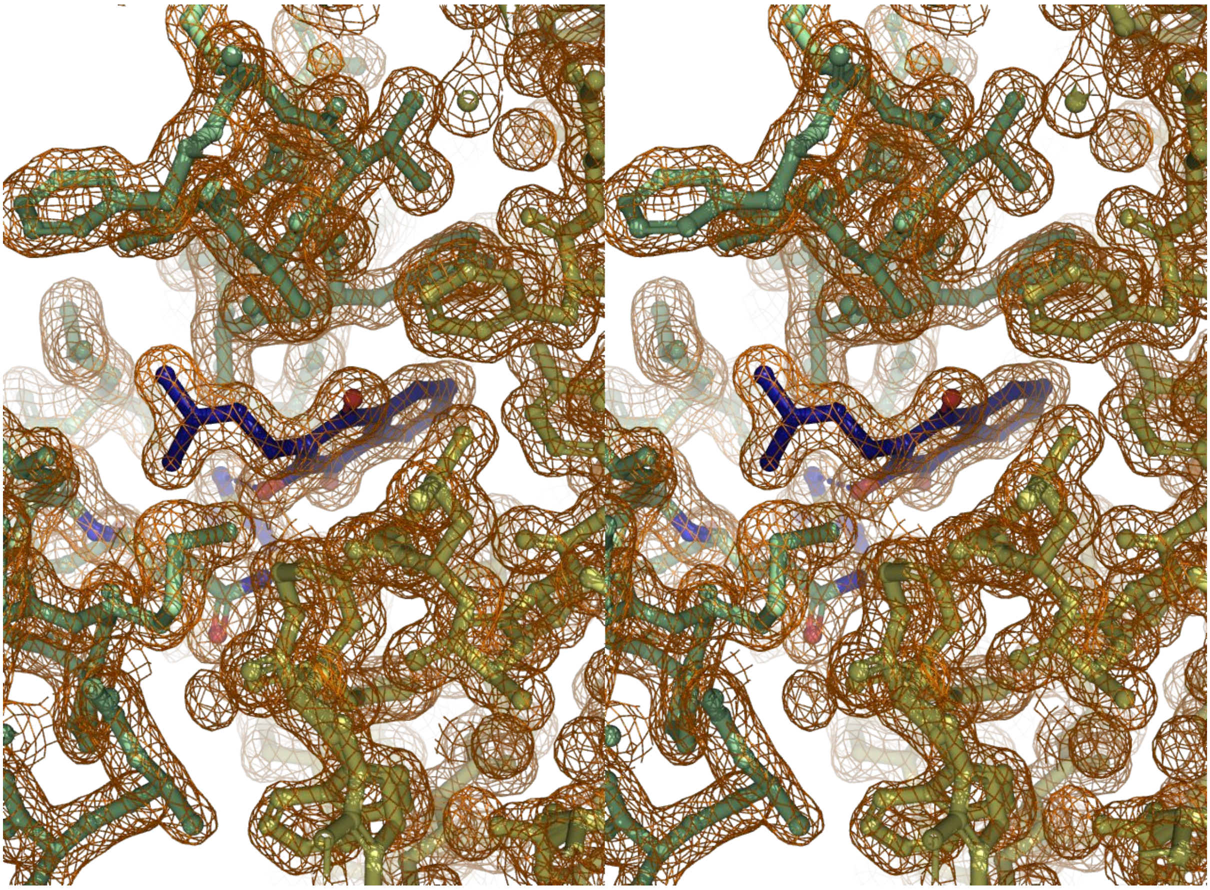

Supplementary Figure S2: Stereoview for the lapachol binding site. Stereoview of the lapachol binding site in the crystal structure, with residues and the ligand represented as sticks. The inhibitor is colored by atoms: oxygen (red), nitrogen (blue), and carbon blue. Protein is colored yellow (C-terminal) or green (N-terminal). The refined 2mFo-DFc electron density map contoured at 1.0 RMSD is shown within 1.6 Å from any atom of protein and ligand.

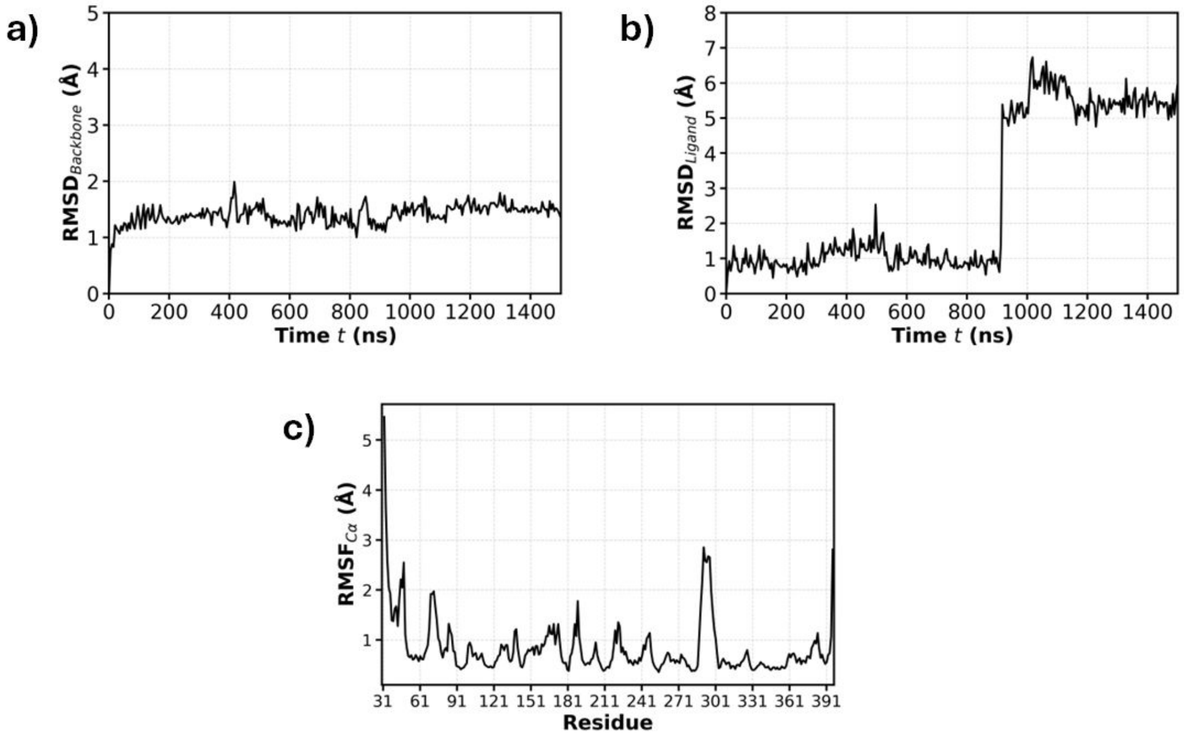

Supplementary Figure S3: RMSD and RMSF analysis of MD simulations for replicate 3. a) RMSD analysis of the *Hs*DHODH backbone protein in complex with lapachol. b) RMSD analysis of lapachol bound to the *Hs*DHODH binding site for replicate 3. c) RMSF (Ca) analysis of the *Hs*DHODH protein in complex with lapachol.

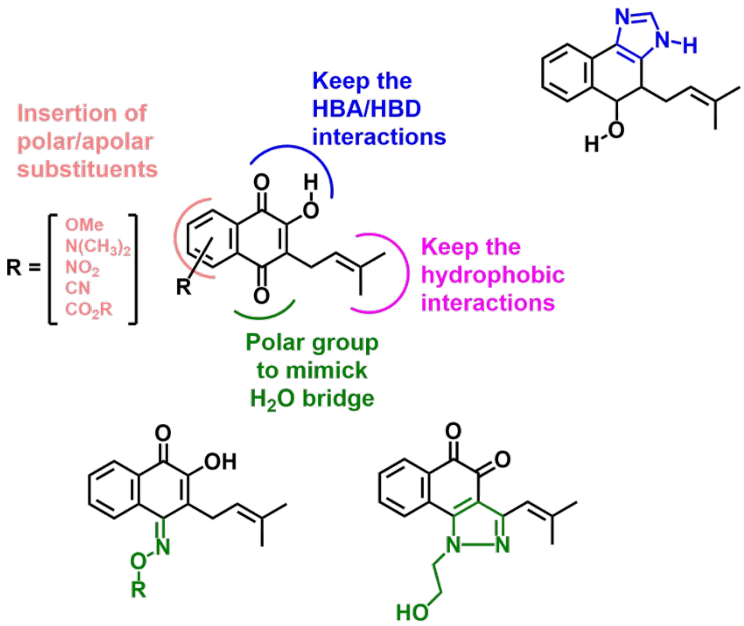

Supplementary Figure S4: Proposed lapachol analogues. Proposed modifications of lapachol molecule that could be synthesized in further projects.
